# Advancing healthcare system performance in Peru through data analytics

**DOI:** 10.3389/fpubh.2026.1849995

**Published:** 2026-08-13

**Authors:** Edmundo Lizarzaburu, Julio Quispe, Macarena Lizarzaburu, Fausto Lizarzaburu, Gabriela Cerna

**Affiliations:** 1 Esan University, Lima, Peru; 2 Monash University, Melbourne, VIC, Australia; 3 Universidad de Lima, Lima, Peru; 4 Pontificia Universidad Catolica del Peru, Lima, Peru

**Keywords:** digital health, health system performance, healthcare analytics, Peru, public health

## Abstract

The utilization of data analytics as an approach to improvement in healthcare system performance through the support of evidence-based decisions, predictive epidemiology, operational efficiency, and equitable distribution of health care resources is becoming increasingly significant. Emerging economies such as Peru are experiencing an increased number of opportunities due to the introduction of digital health initiatives and the implementation of health information systems to improve health service delivery. Evidence related to digital health and health information systems is not fully developed or adequately synthesized with respect to the Peruvian context. Thus, the objective of this study is to generate a Systematic Literature Review (SLR) using PRISMA 2020 Guidelines to describe the contributions of data analytics to improving Healthcare System Performance in Peru, as well as to identify opportunities, obstacles, and future directions of healthcare data analytics in Peru. A systematic search of literature was conducted through Scopus, Web of Science, PubMed/MEDLINE, Scielo, and LILACS using a search strategy that included the following key search terms: Digital Health, Healthcare Analytics, Health Information Systems, Predictive Epidemiology and Data-Driven Healthcare Interventions and was limited to articles written in English or Spanish that were published within the time frame of between 2015 and 2026. Peer-reviewed studies were included when they addressed digital health, healthcare analytics, health information systems, predictive epidemiology, and data-driven healthcare interventions relevant to, or transferable to, the Peruvian healthcare system. The studies selected were based on PRISMA processes for identification, screening, eligibility, and inclusion; the results are synthesized and reported using thematic analysis. The findings of the review show that healthcare analytics has positive effects on disease control, optimization of hospital performance, access to health care, digital governance, and decision-making based on data in public health. However, numerous structural barriers still exist, such as lack of interoperability between fragmented information technology systems, disparity in digital infrastructure between rural and urban areas, regulatory and policy gaps, and disparities in access to health care between rural and urban areas. Ultimately, this review concludes that there is great potential for improvement of Healthcare System Performance in Peru using data analytics, however, in order for there to be sustainable changes, there needs to be continued investment into governance, interoperability between digital ecosystems, ethical management of data, and the development of analytical skills and infrastructure. Future research on healthcare data analytics should be aimed at developing empirical evaluations and context-specific implementation strategies to promote equitable and resilient health care transformation.

## Introduction

Digitalization of health systems around the world strongly affects the way health services are designed, delivered, and assessed. The development of data analytics, artificial intelligence, health information systems, and predictive modeling has opened doors to improving clinical outcomes through enhanced operational processes, epidemiological surveillance, and evidence-based decision-making within healthcare organizations. The rise of digital health records (DHRs) and advanced analytical technologies has allowed healthcare organizations to transition from descriptive methods of care delivery toward creating predictive and preventive health models that enhance both operational efficiency and patient service quality. Over the past several decades, healthcare systems have been challenged by increased demographic demands, limited resources, and increased patient complexity. Implementing data analytics as a strategic asset will improve health systems’ ability to function and adapt, and maintain their role as a sustainable resource.

The application of analytics to the health care sector has been shown through international research to result in enhanced disease surveillance, hospital management, patient outcomes and use of health care resources, as well as enabling the creation of more responsive public health systems. After facing the recent global health crises caused by terrorist attacks and other major disasters; the limitations of fragmented health information systems; slow, methodical decision-making during an emergency response; and unequal access to health services, the need for using analytics in health care is now being recognized as a major priority for health care providers. Therefore, many organizations are now embracing digital transformation initiatives designed to create interoperability, create robust governance structures, ensure ethical use of data, and ensure that institutions are ready to implement sustainable solutions. Although technology will continue to advance globally, the implementation and results from that technology will vary greatly due to differences in country infrastructure, regulatory environment, health workforce capacity, and institutional maturity.

The advent of digital health technology has been elevated on a high priority list for many healthcare providers, as well as policymakers in the country of Peru. There is increasing recognition among both healthcare providers and policymakers in Peru that the integration of digital health technology into the overall healthcare delivery system will enable the development of more robust performance measurement systems, enhance access to care, and reduce the disparity of health care delivery experienced by rural populations. Recent initiatives to enhance health data collection and monitoring through digital technologies, as well as implementing interoperable data collection systems and utilizing analytic tools to drive decision making in healthcare delivery, have all demonstrated the effectiveness of utilizing analytic tools to alleviate some of the issues associated with inefficient operations and to support evidence-based management of care. However, despite these advances being realized through recent digital health initiatives, the healthcare delivery system in Peru continues to struggle with many of the same structural issues that hinder the use of healthcare analytics and digital technology within the healthcare delivery system. Among these structural issues are fragmented data systems, unequal access to digital technology, low levels of analytic capacity among healthcare providers, low levels of governance and oversight of the use of digital technologies, and a lack of sufficient integration between healthcare providers and government agencies. These issues have created an enormous amount of variability in how care is delivered to patients in the Peruvian healthcare delivery system and has limited the ability of healthcare providers to convert digital health innovation into demonstrable improvements in patient health status.

Although there is an increasing amount of interest around digital health transformation, as well as increased interest around analytic techniques within the health sector, the academic literature associated with Peru is largely fragmented and lacks a consolidated synthesis; as such there is no way of knowing the primary themes in Peru related to digital health transformation, the barriers to implementation of digital health technologies, and the priorities that can be defined through the evidence base to support future growth of the healthcare system in Peru. Most of the research published to date consists of individual case studies on specific technologies, or focuses on institutional experiences, or presents discussions of theoretical concepts associated with Digital Health Transformation; therefore, none of the research published to date have provided an integrated understanding of how the use of analytics will eventually improve performance for the overall health service delivery systems. Furthermore, previous reviews have overwhelmingly taken a descriptive approach and have not presented detailed methodologies regarding the steps taken to search for studies, and how studies were selected and synthesized, which has hindered researchers from replicating these reviews and thus made them less useful for contributing to policy and managerial decision-making processes.

In response to this need, the authors of this study conducted a systematic literature review (SLR) based upon PRISMA 2020 guidelines, which aim to bring all available evidence together to create on-going access to a comprehensive pool of information regarding data analytics and its implications for improving the performance of the Peruvian Health System. This research effort has been undertaken as such, providing an integrated and systematic method for accumulating knowledge and creating a database of sources for healthcare researchers. As part of this process, we will establish how data-driven analytics contribute to the enhancement of health care efficiency, predictive epidemiology, digital governance and the improvement of health care accessibility and institutional capacity building. The authors of this study will also assess potential implementation challenges and opportunities regarding the integration of data-driven healthcare systems, as well as potential benefits to Peruvian citizens through its use.

The research in this paper adds to the existing research in three different ways; First is the summarization of the current state of evidence regarding Healthcare Analytics in Peru in a structured, transparent, and reproducible manner using a systematic review protocol. Second is the creation of a thematic framework that groups the literature into two broad categories, i.e., Performance Improvement Mechanisms and Implementing Barriers, and lastly, the article provides policymakers and managers implications that may facilitate future Digital Health Strategic Initiatives and strengthening of the transition to Resilient, Equitable, and Data Driven Health Care Systems in Peru.

## Methodology

A systematic literature review was conducted with methodology described by the PRISMA 2020 (Preferred Reporting Items for Systematic Reviews and Meta Analyses) as a guide to ensure that the systematic review process can be replicated, is transparent, and adheres to a high standard of methodological rigor. The systematic literature review method was chosen for this study because it is a structured way of synthesizing the current body of scientific research and helps to eliminate the potential for subjective biases when identifying, selecting, and interpreting prior research studies. The use of explicit processes for the retrieval, screening, eligibility assessment, and synthesis of research information allows systematic reviews to provide a more trustworthy understanding of how data analytics affect the performance of the Peruvian health care system.

The review process was conducted through a systematic search of five academic databases recognized for their coverage of healthcare, public policy, and information systems research: Scopus, Web of Science, PubMed/MEDLINE, Scielo, and LILACS. These databases were selected to ensure broad international and regional representation while incorporating evidence generated in Latin America and Peru. The search included studies published between 2015 and 2026, reflecting the period during which digital health transformation and healthcare analytics experienced substantial expansion globally and regionally. Publications written in English and Spanish were considered in order to maximize contextual relevance and reduce language related exclusion.

To identify relevant studies, a structured search strategy was developed using combinations of keywords and Boolean operators associated with healthcare analytics and the Peruvian healthcare environment. The search equation was designed iteratively after preliminary exploration and refinement across databases and was defined as follows:

*(“health analytics” OR “data analytics” OR “digital health” OR “health information systems” OR “predictive epidemiology”) AND (Peru OR Peruvian) AND (“healthcare performance” OR “public health” OR hospital OR “health system”)*

The eligibility criteria were established before the screening process to minimize selection bias. Studies were included if they met the following conditions: (1) published in peer reviewed journals; (2) focused on healthcare analytics, digital health, health information systems, artificial intelligence, predictive models, or data driven healthcare interventions; (3) analyzed applications relevant to Peru or generated findings transferable to the Peruvian healthcare context; (4) reported empirical findings, systematic reviews, conceptual frameworks, or implementation experiences; and (5) were available in full text. Studies were excluded when they consisted of editorials, conference abstracts, opinion papers, duplicate records, studies unrelated to healthcare applications, articles without sufficient methodological information, or publications whose full text could not be accessed.

Using the four steps outlined in the (PRISMA 2020) as the guidelines for selecting the appropriate studies, the four steps are: Identification, Screening, Eligibility Assessment, and, Final Inclusion. The first step of the process is the Identification of records, where all records collected from the various databases were exported into a single location. All duplicate records were deleted before the titles and abstracts were screened for relevance. All records that met the eligibility criteria were reviewed in their entirety against the developing criteria. The studies that met these criteria were selected based on their Methodological Relevance and their Direct Connection to the Research Question. A PRISMA Flow Diagram was created to show the progression of the studies through these four steps and to provide transparency for the Review Process.

Each included study was assessed using five criteria: clarity of objectives, methodological transparency, contextual relevance, analytical rigor, and contribution to healthcare system performance. Each criterion was scored as 0 when it was not met and 1 when it was met, producing a total score ranging from 0 to 5. Studies scoring 4–5 were classified as high quality, studies scoring 3 as moderate quality, and studies scoring 0–2 as low quality. The quality scores were used to support the interpretation of the findings.

The creation of thematic categories resulting from the synthesis process describes the primary ways that the use of healthcare analytic tools will improve the performance of a healthcare system. Healthcare analytic tools’ contributions include Predictive Epidemiology and Disease Surveillance, Hospital Efficiency and Operational Optimisation, Healthcare Access and Equity, Governance and Interoperability, and Institutional Capabilities for Digital Transformation. These thematic categories will provide guidance as to how the analysis and findings are discussed later in this Review.

Of the 13 included studies, 11 were classified as high quality, 2 as moderate quality, and none as low quality.

Table 1 summarizes the characteristics of the studies included in the final qualitative synthesis. The reviewed literature encompasses empirical studies, systematic reviews, conceptual frameworks, policy analyses, and implementation experiences addressing healthcare analytics, digital health transformation, health information systems, governance, interoperability, and healthcare performance improvement in Peru and comparable contexts.

**Table 1:** Characteristics of the studies included in the systematic literature review.

References	Country/region	Study type	Main topic	Key findings	Quality score
Wang and Kung (6)	Global	Systematic Review	Healthcare Analytics	Analytics improved operational efficiency, decision-making, and healthcare quality.	5
Vezyridis and Timmons (7)	United Kingdom	Critical Review	Data Governance	Governance and public trust are essential for digital health adoption.	4
Khanra et al. (8)	Global	Systematic Review	Big Data Analytics	Big data analytics enhances hospital performance and patient outcomes.	5
Shilo et al. (9)	Global	Empirical Study	Digital Health	Digital technologies strengthened pandemic response and monitoring systems.	4
Allin et al. (1)	Global	Conceptual Study	Digital Health Equity	Digital divide remains a major challenge for equitable healthcare access.	3
Carrillo-Larco et al. (2)	Peru	Review Study	Health System Development	Peru has made progress in health innovation but faces structural challenges.	4
Rodríguez et al. (10)	Peru	Empirical Study	Rural Digital Health	Infrastructure limitations restrict digital health implementation in rural areas.	4
Raimundo and Quintana (11)	Latin America	Policy Analysis	Digital Transformation	Regional policy frameworks are essential for sustainable digital health systems.	3
Elragal and Haddara (5)	Global	Literature Review	Healthcare Analytics	Analytics supports evidence-based management and resource optimization.	4
Paul and Osei-Frimpong (12)	Global	Review Study	Data Privacy and Security	Privacy and cybersecurity remain key adoption barriers.	4
Gutiérrez-Aguado et al. (13)	Peru	Case Study	Data Science Education	Capacity-building initiatives are essential for healthcare analytics adoption.	4
El-Khoury et al. (4)	Global	Review Study	AI Governance	Ethical AI deployment requires stronger regulatory and governance mechanisms.	4
Díaz-Obregón et al. (3)	Peru	Empirical Study	Health Research Priorities	Data-driven approaches support evidence-based planning in EsSalud.	4

Of the 13 studies included in the review, seven addressed global contexts, four focused specifically on Peru, one examined Latin America, and one focused on the United Kingdom. Eleven studies were published from 2020 onward. Governance, privacy, and interoperability represented the most frequent thematic area, accounting for 30.8% of the included studies.

## Results

**Figure 1:**
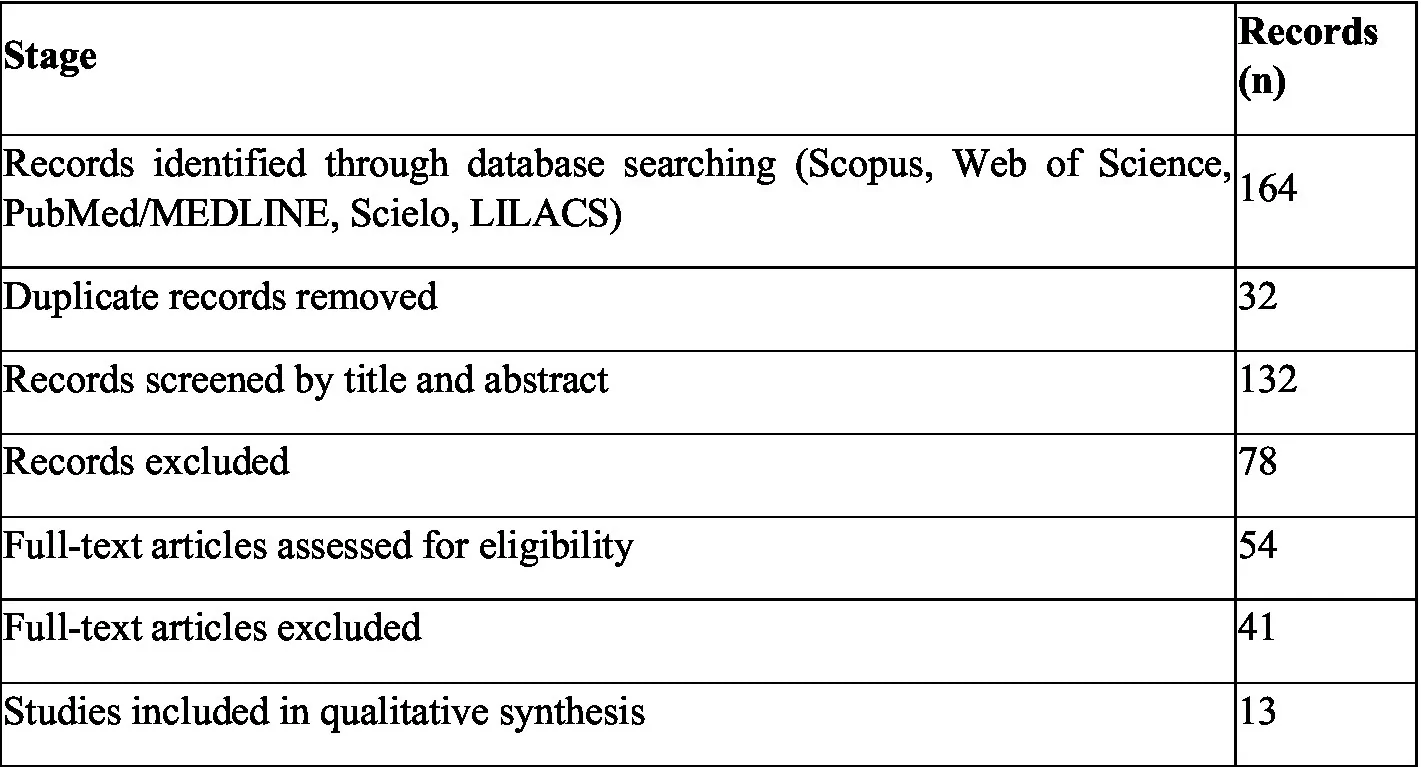
PRISMA 2020 study-selection summary showing the identification, screening, eligibility assessment, and final inclusion of studies in the systematic literature review.

The database search identified 164 records. After removing 32 duplicate records, 132 articles remained for title and abstract screening. Following the screening process, 54 full-text articles were assessed for eligibility. Forty-one studies were excluded because they did not meet the predefined inclusion criteria, resulting in a final sample of 13 studies included in the qualitative synthesis. The complete study selection process is presented in Figure 1.

The initial study pool was established through the systematic search process using the databases Scopus, Web of Science, PubMed/MEDLINE, Scielo and LILACS. After removing duplicate records, the remaining articles were screened based on their titles and abstracts against pre-defined inclusion/exclusion criteria. Studies deemed to be relevant based on these assessments were reviewed in their entirety, and only those studies that were determined to be directly applicable to one (or more) of the areas of interest for this review—Healthcare Analytics, Digital Health, Health Information Systems and Data Driven Interventions Related to Healthcare in Peruvian Context, were included in the final synthesis. This systematic selection process for the articles was documented according to the PRISMA 2020 flowchart guidelines to ensure a clear understanding and repeatability of the processes involved in producing this review.

Throughout the research time period, there was continued growth in the number of scientific articles published in the area of healthcare analytics and digital transformation within the healthcare systems. The majority of publications were produced after 2020, likely due to the rush to adopt these new forms of technology at an accelerated rate following increased interest from health organizations in building resilient public health systems, creating improved monitoring mechanisms for infectious disease outbreaks and modernizing healthcare delivery models. The body of literature identified during this research project consisted of a majority of published empirical research, systematic reviews, theoretical analysis, implementation reports and policy-related research that focused on building digital health ecosystems. While the vast majority of the literature reviewed during the project continues to focus on international evidence, an increasing amount of research is being published regarding the issues and opportunities related to Peru and Latin America.

The findings were synthesized to delineate five major themes through which data analytics impact healthcare performance. The first theme addressed by the data analysis and subsequent synthesis is predictive epidemiology and disease surveillance. The evidence indicates that the use of analytical models in conjunction with digital monitoring tools enhances healthcare systems’ ability to forecast disease trends, improve the early warning mechanisms of a system, and improve public health interventions. Additionally, predictive methodologies enhance the efficiency of how healthcare resources are allocated, and improve health system responsiveness during times of health emergency. Predictive methodologies accomplish this by transforming both current and historical information into actionable information.

Analytics driven technology helps both healthcare organizations and individual employees perform better from an operational standpoint through improved performance and operational efficiency in regards to patient flow and scheduling, along with improving the ability to monitor healthcare quality indicators as well as maximizing the efficient use of infrastructure and personnel resources. Based upon multiple studies, the use of analytics driven management provides opportunities for the reduction of inefficiencies and improvement of the quality of care provided within the healthcare industry when integrated into an overall digital transformation strategy.

The third theme relates to healthcare accessibility and equity for underserved populations, pointing out the potential of digital analytics to reduce information inequities and support equitable healthcare coverage. However, the evidence suggests the advantages are still not distributed evenly due to a lack of infrastructure, differing levels of digital readiness, and varying institutional capabilities among jurisdictions. Barriers to implementing effective and meaningful analytics still exist for rural and remote areas (see Table 2).

**Table 2:** Distribution of included studies by main theme.

Main theme	Number	Percentage
Predictive epidemiology and surveillance	1	7.7%
Hospital efficiency and healthcare analytics	3	23.1%
Healthcare access and equity	2	15.4%
Governance, privacy, and interoperability	4	30.8%
Institutional capacity and digital transformation	3	23.1%
Total	13	100%

The governance, interoperability, and ethical utilization of health data fall within the governance category of the review paper’s four thematic bases. As such, this paper has shown that success in implementing healthcare analytics is not only reliant upon the adoption of technology, but also upon the coordination of institutions, the regulatory maturity of the institutions using the technology, the cybersecurity practices of the institutions using the technology, and the safeguards established by institutions to protect the privacy of the consumer and to use the data in a responsible way. The major challenge of thematic area four is interoperability, which is particularly problematic in healthcare systems that are fragmented, or in which multiple healthcare providers maintain separate information systems that do not communicate with one another.

The review also revealed that having sufficient institutional and human capacity for digital transformation will likely be an important enabling condition for the successful implementation of sustainable healthcare analytics. Results from the literature indicate that the use of analytical technologies can lead to increased value when there are efforts to develop the skills of the workforce, promote organizational learning, and invest in the development of data literacy by healthcare professionals and decision-makers. Furthermore, the literature repeatedly states that developing institutional analytical capacity is essential to the ability to convert technological investments into measurable improvements in health care quality.

In summary, findings of the research show that healthcare systems in Peru use data analytics as a means to enhance organizational performance through improving their operational efficiency and improving their ability to support epidemiological surveillance and to improve the quality of clinical decisions through evidence-based decision-making processes. Through data analytics, health systems also have access to more equitable healthcare opportunities and services for patients. The long-term success of working with data analytics will depend on addressing the ongoing structural barriers to data governance; infrastructure; interoperability; and institutional readiness.

## Discussion

Based on the analysis conducted during the course of this systematic literature review, there is increasing evidence that data analytics is no longer simply a technological support tool but rather has become an integral component of the strategic capabilities required for enhancing the performance of healthcare systems. This data from the literature indicates that analytical technology improves not just operational efficiencies and epidemiological surveillance, but also helps organizations achieve a mission that is more aligned with institutional goals regarding improved healthcare access, governance, quality, and sustainability of the healthcare system over the longer term. Furthermore, these findings are consistent with the international literature identifying data-driven healthcare systems as a critical enabler of modernizing the public sector and transforming the healthcare industry today. While much has been published regarding data analytics, the research reviewed in this systematic literature review provides strong evidence that simply having access to such technology does not guarantee improved outcomes within the healthcare industry. Instead, the processes by which organizations implement and integrate new technologies into their respective organizations is heavily dependent upon existing organizational and institutional structures and processes.

The findings within Peru indicate that fragmented structures limit the capacity for widespread adoption and sustainability of Digital Health projects. Despite the gradual shift to Implement Information Systems, Predictive tools, and Digital Health in many Peruvian Health Institutions, the distribution of implementing these elements between various territories and institutions remains uneven. This reflects a larger trend among Emerging Economies (the same as in Peru) where while new technologies are becoming available to certain organizations, they are still being utilized by organizations that have greater levels of infrastructure and administrative capacity, creating a disparity between the level of access to digital health benefits between rural and underserved populations compared to larger urban centers.

The main finding of this review is the significance of governance and interoperability in evaluating a healthcare’s analytic performance. The studies reviewed in this article consistently stated that a fragmented environment for information significantly limits analytical systems’ ability to create actionable outputs, i.e., they provide little evidence for making data-driven decisions. Interoperability challenges do not occur in isolation but represent a more extensive issue with governance. Governance challenges typically include institutional coordination, regulation alignment, data standardization, and accountability. Therefore, the findings of this review recommend that any government healthcare modernization efforts should promote integrated digital ecosystems and relationships between institutions, rather than simply investing in technology infrastructure alone.

The review also demonstrates that healthcare equity remains one of the most critical dimensions shaping the future impact of data analytics in Peru. Although analytical tools create opportunities to improve disease monitoring, optimize resource allocation, and expand healthcare access, existing inequalities may limit these benefits if implementation strategies do not explicitly address territorial and social disparities. This observation reinforces arguments from recent digital transformation literature indicating that technological innovation can unintentionally reproduce existing inequalities when institutional capacities and access conditions remain uneven. Therefore, healthcare analytics should be understood not only as a technical intervention but also as a governance and inclusion strategy.

Another relevant contribution of this review concerns the importance of institutional analytical capabilities and workforce readiness. The evidence suggests that organizations generating stronger outcomes through healthcare analytics are not necessarily those with the most advanced technologies but those capable of integrating analytical outputs into managerial and clinical decision processes. Data literacy, interdisciplinary collaboration, organizational learning, and continuous training emerged as recurrent enabling factors supporting successful implementation. This finding highlights the importance of investing in human capital and institutional adaptation alongside digital infrastructure development.

From a policy perspective, the findings indicate that advancing healthcare system performance through analytics in Peru requires coordinated action across multiple dimensions. Priority areas include strengthening interoperability standards, establishing clearer governance frameworks for ethical data use, expanding digital infrastructure in underserved regions, and promoting analytical competencies across healthcare institutions. Public investment strategies should emphasize scalability and integration while creating mechanisms capable of translating analytical insights into operational and policy improvements.

Finally, this study contributes to the literature by moving beyond isolated discussions of digital health technologies and providing a structured synthesis of the mechanisms through which analytics influences healthcare system performance. Nevertheless, the review also reveals important gaps in existing knowledge. Future research should prioritize empirical evaluation of implemented analytics programs, comparative studies across regions, longitudinal analyses of healthcare performance outcomes, and stronger evidence regarding the social and economic impacts of digital transformation initiatives in Peru.

## Conclusion

This study conducted a Systematic Literature Review (SLR) following PRISMA 2020 guidelines to synthesize existing evidence regarding the contribution of data analytics to healthcare system performance in Peru. The findings demonstrate that healthcare analytics has emerged as a relevant driver of healthcare transformation by improving decision making processes, strengthening predictive epidemiology and disease surveillance, optimizing hospital operations, and supporting more efficient allocation of healthcare resources. Across the reviewed literature, analytics appears not only as a technological instrument but also as an enabling mechanism for strengthening institutional responsiveness and promoting more evidence based healthcare management.

The review further shows that the benefits generated by healthcare analytics remain strongly conditioned by contextual and institutional factors. Persistent challenges associated with fragmented information systems, unequal digital infrastructure, limited interoperability, governance constraints, and disparities between urban and rural healthcare environments continue to restrict the scalability and sustainability of digital health initiatives in Peru. These findings suggest that technological adoption alone is insufficient to generate meaningful improvements in healthcare outcomes unless accompanied by institutional coordination, workforce readiness, and long term investment in digital capabilities.

From a practical perspective, the study highlights the importance of developing integrated digital ecosystems capable of supporting secure data exchange, strengthening governance mechanisms, and improving institutional analytical capacity. Policies oriented toward healthcare modernization should prioritize interoperability standards, ethical management of health data, equitable access to digital infrastructure, and capacity building among healthcare professionals and decision makers. Advancing these dimensions may contribute to transforming healthcare analytics into a sustainable instrument for improving healthcare accessibility, quality, and resilience.

This review also contributes to the literature by providing a transparent and reproducible synthesis of evidence through a systematic methodological approach and by organizing current knowledge into thematic domains that explain how analytics influences healthcare system performance. By focusing specifically on the Peruvian context while integrating broader international evidence, the study offers a foundation for future research and policy design in digital health transformation.

Despite these contributions, several limitations should be acknowledged. The review was restricted to selected academic databases and publications available in English and Spanish, which may have excluded relevant grey literature or local implementation experiences. In addition, differences in study designs and healthcare contexts limited direct comparison across findings. Future research should prioritize empirical assessments of implemented analytical initiatives, longitudinal evaluations of healthcare outcomes, regional comparative analyses, and deeper examination of governance and equity dimensions associated with digital health adoption in Peru.

Overall, data analytics represents a significant opportunity to strengthen healthcare system performance in Peru; however, realizing its full potential will depend on the capacity of institutions to integrate technological innovation with governance, inclusion, interoperability, and sustained organizational transformation.
